# Aliasing mitigation in optical microscopy of dynamic biological samples by use of temporally modulated color illumination and a standard RGB camera

**DOI:** 10.1117/1.JBO.25.10.106505

**Published:** 2020-10-26

**Authors:** Christian Jaques, Michael Liebling

**Affiliations:** a Idiap Research Institute, Martigny, Switzerland; b École Polytechnique Fédérale de Lausanne, Lausanne, Switzerland; c University of California Santa Barbara, Department of Electrical and Computer Engineering, Santa Barbara, California, United States

**Keywords:** generalized sampling, B-splines, computational imaging, microscopy, spectral unmixing

## Abstract

**Significance:** Despite recent developments in microscopy, temporal aliasing can arise when imaging dynamic samples. Modern sampling frameworks, such as generalized sampling, mitigate aliasing but require measurement of temporally overlapping and potentially negative-valued inner products. Conventional cameras cannot collect these directly as they operate sequentially and are only sensitive to light intensity.

**Aim:** We aim to mitigate aliasing in microscopy of dynamic monochrome samples by implementing generalized sampling via the use of a color camera and modulated color illumination.

**Approach:** We solve the overlap problem by spectrally multiplexing the acquisitions and using (positive) B-spline segments as projection kernels. Reconstruction involves spectral unmixing and inverse filtering. We implemented this method using a color LED illuminator. We evaluated its performance by imaging a rotating grid and its applicability by imaging the beating zebrafish embryo heart in transmission and light-sheet microscopes.

**Results:** Compared to stroboscopic imaging, our method mitigates aliasing with performance improving as the projection order increases. The approach can be implemented in conventional microscopes but is limited by the number of available LED colors and camera channels.

**Conclusions:** Generalized sampling can be implemented via color modulation in microscopy to mitigate temporal aliasing. The simple hardware requirements could make it applicable to other optical imaging modalities.

## 1 Introduction

Observing phenomena in live biological samples in microscopy requires sufficient time resolution.^1^ In addition to the development of faster and more sensitive cameras and clever pixel rebinning methods,^2^ various sensing and computational approaches to increase the temporal resolution of microscopes have been proposed. Some rely on multiple observations of a signal^3^^–^^6^ or make clever use of the signal structure itself, e.g., its sparsity in a known basis^7^^–^^9^ or its repeatable nature.^10^^,^^11^ The ability to modulate the illumination rapidly in a controlled and cost-effective way (in particular, with LED-based illuminators^12^) also opens the way for promising methods. For instance, short light pulses (stroboscopy) have been used to reduce motion blur,^13^ while the fluttered shutter principle^14^^,^^15^ uses a pseudorandom temporal illumination sequence to computationally improve the temporal resolution.

Despite the above developments, many imaging systems still rely on direct image acquisition, which is vulnerable to aliasing if the imaged signal contains frequencies higher than the Nyquist frequency, since perfect low-pass filters cannot be implemented in practice. Generalized sampling^16^ offers a framework to implement sampling operations that relax the need for ideal filters. Applications that build upon this framework have been proposed in optics^17^ or for sample signals with a finite rate of innovation on multiple channels.^18^

Due to the shift-invariant nature of generalized sampling, the implementation of prefilters in time can be problematic because of the temporal overlap of the inner product kernels. Indeed, if the prefilter used is longer than a unit of time (sampling interval), several inner products must be carried out simultaneously, which requires a multiplexed acquisition approach. Moreover, in standard (incoherent light) optical imaging applications, only the intensity of the light, which is always positive, can be measured. While modulation is possible by illuminating the sample with a variable intensity over time, this illumination suffers from the same positivity limitations (only positive illumination functions can be considered). In this paper, we present an approach to overcome the above positivity and multiplexing limitations to carry out generalized sampling in the context of optical microscopy imaging. We propose to use active multicolor illumination and a color camera for collection, allowing spectral multiplexing and ideal prefiltering of the signal. Specifically, we modulate the illumination signal over time with independent signals in different color channels of the illumination lamps, which produces modulated signals whose integration by a camera shutter can be converted to the inner product between the signal and the prefilter kernel. Following recovery of these coefficients and reconstruction of the signal in the projection basis’s dual basis, we obtain samples of the incoming signal, projected on the space spanned by a shift-invariant B-spline basis.

This paper is based on our previous paper introducing generalized temporal sampling imaging for microscopy.^19^ The rest of the paper is organized as follows. In Sec. 2, we provide a formal description of our problem. In Sec. 3, we derive our method. In Sec. 4, we illustrate our approach on both synthetic signals and from data collected on a transmission wide-field microscope. Finally, we conclude in Sec. 5.

## 2 Problem Statement

We consider a continuous-time signal f(t) for which we want to estimate the least-squares approximation $\overset{˜}{f}(t)$ in the shift-invariant space spanned by B-splines of degree n, $V=\{\beta^{n}(\cdot -k),k\in Z\}$, which can be obtained via a biorthogonal projection^16^

$$\overset{˜}{f}(t)=\underset{k\in Z}{\sum }\underset{c[k]}{\underset{⏟}{\left\langle f,{\overset{{}^{\circ}}{\beta }}^{n}(\cdot -k)\right\rangle }}\beta^{n}(t-k),$$ (1)

with

$$\langle f,g\rangle =\int_{-\infty }^{\infty }f(t)g(t)dt$$ (2)

and where ${\overset{{}^{\circ}}{\beta }}^{n}(t)$ is the dual B-spline,^20^ a function which, in addition to satisfying,

$$\langle \beta^{n}(\cdot -\ell ),{\overset{{}^{\circ}}{\beta }}^{n}(\cdot -k)\rangle =\delta [\ell -k],$$ (3)

also spans the subspace V (and is, therefore, unique).

A practical system should thus provide the measurements $c_{k}$, which correspond to weighted integrals of the input signals. There are two issues when implementing this. First, the ${\overset{{}^{\circ}}{\beta }}^{n}$ has infinite support and hence overlap in time (except for degree n=0), which requires some way of splitting the input signal to perform the inner products in parallel, and second, the signals in this setting are light intensities, which have to be positive.

To implement the projection settings, we consider a conventional imaging system with a camera that has C color channels (e.g., an RGB pixel of a color camera corresponds to C=3). We further assume that the system has L light sources that uniformly illuminate the scene, each with a different, yet possibly overlapping, color spectrum. The intensity of each light source can be independently controlled as a function of time. These functions can only be positive (as light intensities are necessarily positive) and their duration should be less than the integration time of each of the camera’s frames. We model the imaging system by taking into account the crosstalk that arises from the use of broadband light sources and wide camera (RGB) filters. For a single pixel at time k, we simultaneously measure the intensities $y_{c}[k]$ in channels c=1,…,C

$$\left(\begin{array}{c} y_{1}[k]\\ \vdots \\ y_{C}[k] \end{array})=(\begin{array}{cccc} \gamma_{1,1} & \cdots & \gamma_{1,L} & d_{1}\\ \vdots & \ddots & \vdots & \vdots \\ \gamma_{C,1} & \cdots & \gamma_{C,L} & d_{C} \end{array})(\begin{array}{c} a_{1}[k]\\ \vdots \\ a_{L}[k]\\ 1 \end{array}\right)$$ (4)

or in condensed form

y=Γa, (5)

where the matrix Γ contains the crosstalk mixing terms (including an affine offset) and the coefficients $a_{\ell }[k]$ are the inner products

$$a_{\ell }[k]=\langle f,s_{\ell }(\cdot -k)\rangle ,$$ (6)

where $s_{\ell }(t)$ are the positive illumination functions, whose support covers the sensor exposure interval.

With this formulation, the problem of recovering the samples c[k] in Eq. (1) from measurements $y_{c}[k]$ can be broken down into the following subproblems:

1. Find suitable illumination functions $s_{\ell }$ (in particular, positive and with a finite support) such that the sequence c[k] can be derived from the multichannel sequence $a_{\ell }[k]$.
2. Determine the coefficients c[k] from the coefficients $a_{\ell }[k]$ (via inverse filtering).
3. Determine the coefficients $a_{\ell }[k]$ from the $y_{c}[k]$ (via spectral unmixing).

We detail these steps in the section below.

## 3 Methods

### 3.1 Multicolor B-Spline Segments as Prefilter Kernels

**Fig. 1:**
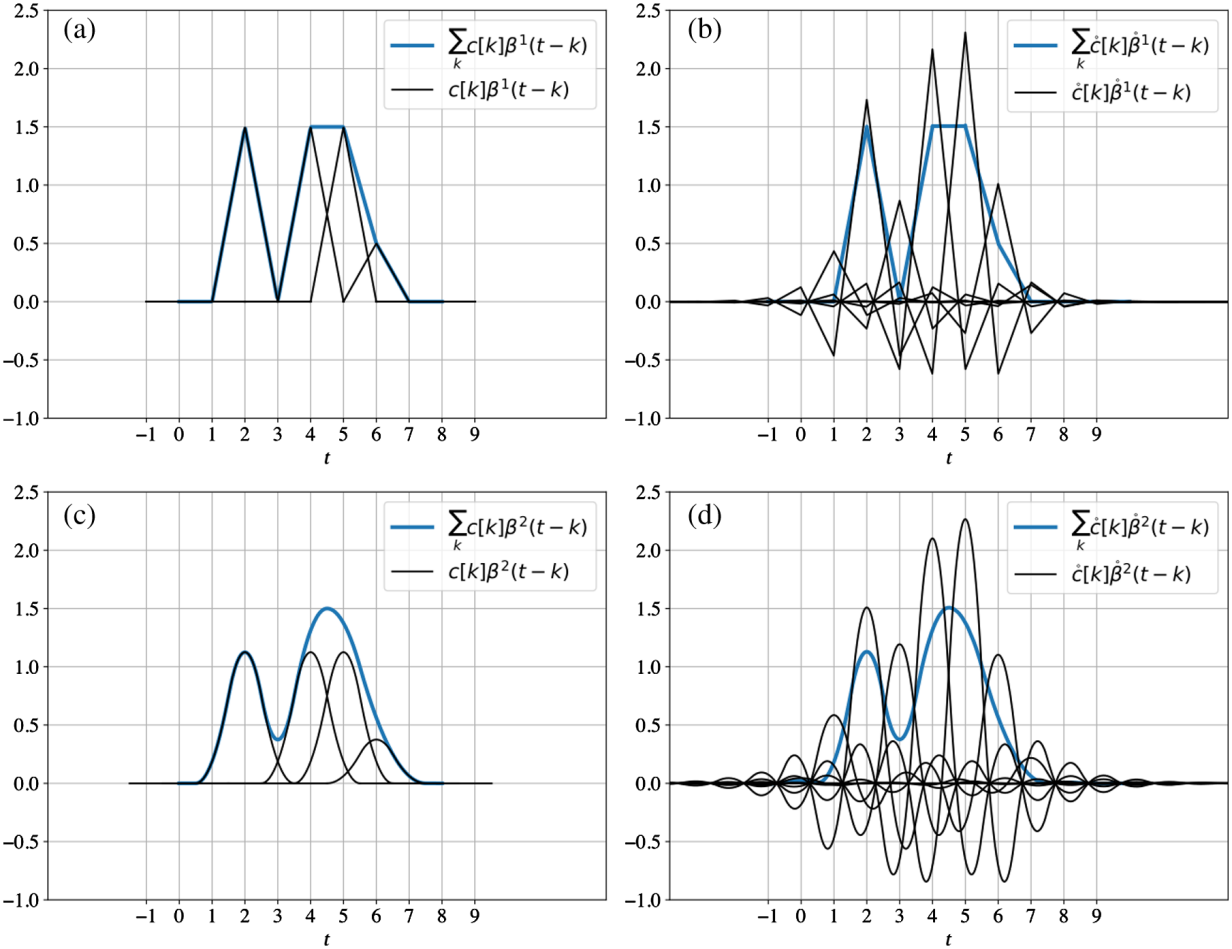
Equivalent basic and dual B-spline representations of the same signal for degrees (a), (b)n=1 and (c), (d) n=2. Notice that both represented signals (in blue in all plots) are equivalent, i.e., $\underset{k}{\sum }c[k]\beta^{n}(t-k)=\underset{k}{\sum }\overset{{}^{\circ}}{c}[k]{\overset{{}^{\circ}}{\beta }}^{n}(t-k)$, while the basic B-spline representation uses functions of finite support.

Our goal is to compute the inner products in Eq. (1) via active illumination by appropriately choosing illumination functions $s_{\ell }(t-k)$. The challenge of this task is that we cannot use $s_{\ell }(t-k)={\overset{{}^{\circ}}{\beta }}^{n}(t-k)$ directly (which would be the natural choice) because the dual of a B-spline is not positive for all t (except when n=0) and because $s_{\ell }(t)$ a light intensity, it must be positive. We work around this problem using an equivalent representation of the projection described in Eq. (1) and shown in Fig. 1, by switching the role of the dual bases^20^ namely,

$$\overset{˜}{f}(t)=\underset{k\in Z}{\sum }\langle f,{\overset{{}^{\circ}}{\beta }}^{n}(\cdot -k)\rangle \beta^{n}(t-k)=\underset{k\in Z}{\sum }\langle f,\beta^{n}(\cdot -k)\rangle {\overset{{}^{\circ}}{\beta }}^{n}(t-k).$$ (7)

Specifically, the coefficients in the dual B-spline basis are given as

$$\overset{{}^{\circ}}{c}[k]=\langle \beta^{n}(\cdot -k),f\rangle =\int_{-\frac{n+1}{2}}^{\frac{n+1}{2}}\beta^{n}(t-k)f(t)dt,$$ (8)

where we note that all involved functions are positive and, since the B-spline has a finite support, the inner-product can be computed over a finite interval. Nevertheless, since the support of B-splines of degree n is n+1, we have n+1 shifted B-splines that overlap in the signal representation at any given time (see Fig. 1) making the sequential computation of the $\overset{{}^{\circ}}{c}[k]$ problematic.

In order to still acquire several inner products simultaneously, we spectrally multiplex the measurements according to Eq. (4) by splitting each B-spline into n+1 regions, which gives the following illumination functions $s_{\ell }(t)$. For n illumination sources (ℓ=1,…,n); for n=0, we have

$$s_{1}(t)=\beta^{0}(t),$$ (9)

for n=1, we have

$$s_{1}(t)=\{\begin{array}{ll} \beta^{1}(t)=1-t, & 0<t<1\\ 0, & \text{otherwise}, \end{array}$$ (10)

$$s_{2}(t)=\{\begin{array}{ll} \beta^{1}(t-1)=t, & 0<t<1\\ 0, & \mathrm{otherwise}, \end{array}$$ (11)

and for n=2, we have

$$s_{1}(t)=\{\begin{array}{ll} \beta^{2}(t+1)=\frac{1}{2}{\left(\frac{1}{2}-t\right)}^{2} & -1/2<t<1/2\\ 0, & \mathrm{otherwise}, \end{array}$$ (12)

$$s_{2}(t)=\{\begin{array}{ll} \beta^{2}(t)=\frac{3}{4}-t^{2}, & -1/2<t<1/2\\ 0, & \mathrm{otherwise}, \end{array}$$ (13)

$$s_{3}(t)=\{\begin{array}{ll} \beta^{2}(t-1)=\frac{1}{2}{\left(\frac{1}{2}+t\right)}^{2} & -1/2<t<1/2\\ 0, & \mathrm{otherwise}, \end{array}$$ (14)

where

$$\beta^{2}(t)=\{\begin{array}{ll} \frac{1}{2}(\frac{3}{2}-|t|{)}^{2}, & \frac{1}{2}<|t|<\frac{3}{2}\\ \frac{3}{4}-t^{2}, & |t|\leq \frac{1}{2}\\ 0, & \mathrm{otherwise}. \end{array}$$ (15)

**Fig. 2:**
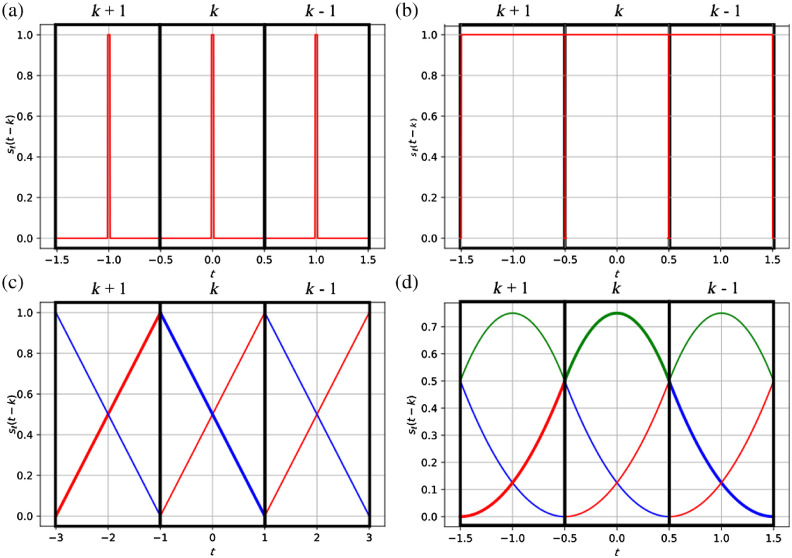
Illumination functions $s_{\ell }(t-k)$ over three frames for stroboscopic imaging (a) n=0, n=1, and (c) n=2. The color of the line corresponds to the color of the illumination light. For (c), (d) n=1 and n=2, multiple frames are involved to compute a single coefficient $\overset{{}^{\circ}}{c}[k]$ from the coefficients $a_{\ell }$.

Figure 2 shows the illumination functions over three consecutive acquired frames for stroboscopic illumination and splines of degrees n=0, n=1, and n=2. The color of the line corresponds to the color of the illumination. Notice that to reconstruct $\overset{{}^{\circ}}{c}[k]$ we have to combine measurements over multiple frames (see the bold lines in Fig. 2 for n=1 and n=2).

The red illumination comes from light source 1 ($a_{1}$), the blue illumination comes from light source 2 ($a_{2}$), and the green illumination from light source 3 ($a_{3}$). For degrees n=0,1, and 2, the coefficients $\overset{{}^{\circ}}{c}[k]$ can be recovered from $a_{\ell }[k]$ as described below (assuming mirror boundary conditions).

Degree n=0:

$$\overset{{}^{\circ}}{c}[k]=a_{1}[k].$$ (16)

Degree n=1:

$$\overset{{}^{\circ}}{c}[k]=\{\begin{array}{ll} a_{1}[k]+a_{2}[k+1], & 0\leq k<K-1\\ 2a_{1}[K-1], & k=K-1 \end{array}.$$ (17)

Degree n=2:

$$\overset{{}^{\circ}}{c}[k]=\{\begin{array}{ll} a_{1}[k-1]+a_{3}[k]+a_{2}[k+1], & 1\leq k<K-2\\ a_{2}[0]+a_{3}[0]+a_{2}[1], & k=0\\ a_{1}[K-1]+a_{1}[K-1]+a_{1}[K-2], & k=K-1 \end{array}.$$ (18)

### 3.2 Spectral Unmixing

The spectral unmixing procedure is similar to the setting described in Jaques et al.^21^ Specifically, given the measurements y and having built d and Γ from calibration, we recover the vector a by solving the minimization problem

$$a^{⋆}=\underset{a}{\min}{\left\|y-d-\Gamma a\right\|}_{2}^{2}.$$ (19)

### 3.3 Converting to Samples

We obtain the basic B-spline representation through filtering of the coefficients in the dual-spline representation^20^

$$c[k]=(b^{2n+1}{)}^{-1}*\overset{{}^{\circ}}{c}[k].$$ (20)

We can further obtain samples by carrying out interpolation

$$\tilde{f}[k]=\tilde{f}(t){|}_{t=k}=c*b_{1}^{n}[k],$$ (21)

where

$$b_{1}^{n}[k]=\beta^{n}(t){|}_{t=k}.$$ (22)

## 4 Experiments

### 4.1 Reconstructions from Synthetic Data

**Fig. 3:**
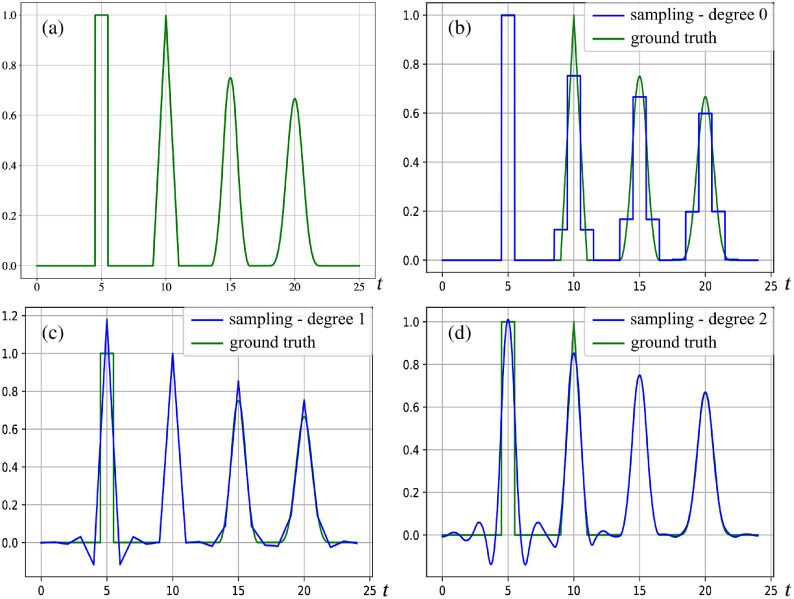
Shifted B-splines of degrees 0, 1, and 2. We observe that each B-spline is perfectly reconstructed when the sampling is done with B-splines of the same degree, i.e., the B-spline of degree 0 in (b) is perfectly sampled, the B-spline of degree 1 in (c) is perfectly sampled, and the B-spline of degree 2 in (d) is perfectly sampled.

We conducted an experiment to illustrate the impact of the chosen sampling basis. We generated a one-dimensional (1-D) signal containing shifted B-splines of various degrees, shown in Fig. 3. We then simulated active sampling using B-splines of degrees 0 to 2 as prefilters. Figure 3 shows the reconstructions obtained by sampling the signal on the top left of the figure. We observe that the B-splines in the sampled signal are perfectly reconstructed when the prefilter is of the same degree as the B-spline. We also see overshooting and ringing after sharp transitions, for degrees n≥1

$$\beta^{0}(t-5)+\beta^{1}(t-10)+\beta^{2}(t-15)+\beta^{3}(t-20).$$

**Fig. 4:**
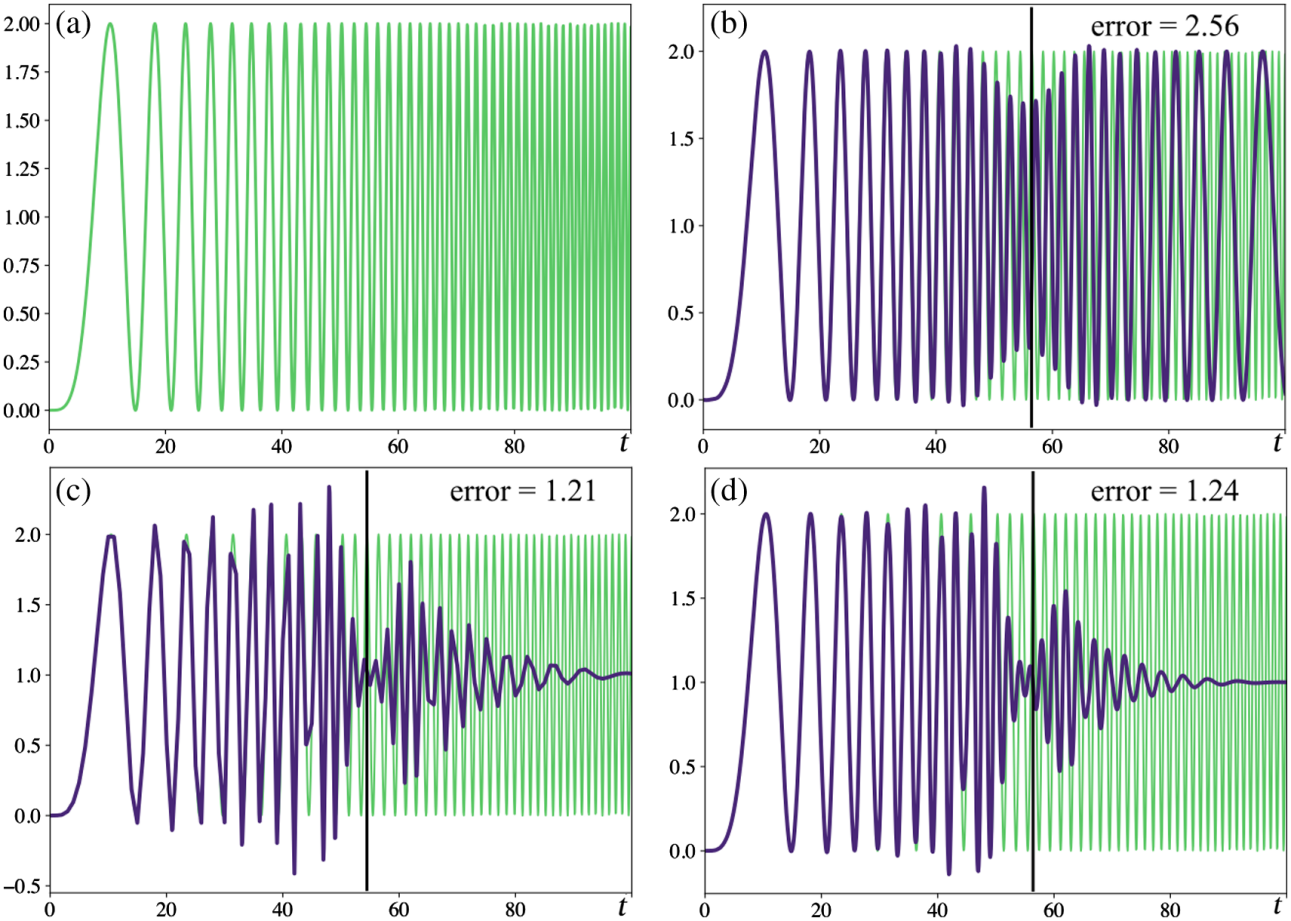
Sampling simulations of the signal in (a) $f(t)=1+\sin(\frac{t^{2}}{35}+\frac{\pi }{2})$. We simulated (b) stroboscopic imaging and generalized sampling with B-splines of (c) degrees 1 and (d) 2. The vertical black line shows time after which the Shannon–Nyquist criterion is no longer respected (frequency too high). For each plot, the reconstruction error energy is shown and stroboscopic has the higher error. Notice that (c), (d) the B-spline sampling reconstructions for high frequencies (t≥80) goes to the average of the signal, while (b) the stroboscopic imaging shows aliasing.

We set out to investigate the effect of our method in the presence of high frequencies, which we compared to a stroboscopic imaging system. We generated a 1-D temporal chirp signal and simulated sampling with B-splines of degree 2 as well as stroboscopic imaging. The signal and sampling simulations are shown in Fig. 4, where we notice that stroboscopic imaging is strongly subject to aliasing, while generalized sampling by projection on B-spline bases gracefully handles higher frequencies. Our chirp function is $f(t)=1+\sin(\frac{t^{2}}{35}+\frac{\pi }{2})=1+\sin(\phi (t))$, with the instantaneous ordinary frequency defined as $\frac{d\phi (t)}{dt}\frac{1}{2\pi }=\frac{t}{35\pi }$. In the simulation, our sampling period is 1, so the Nyquist frequency is 1/2, i.e., the Shannon–Nyquist sampling criterion is not respected anymore when $\frac{t}{35\pi }\geq \frac{1}{2}\Rightarrow t^{⋆}=\frac{35\pi }{2}\approx 55$. In Fig. 4, we show the normalized energy of the error of reconstruction computed on the signal where the Shannon–Nyquist criterion is not respected (after the black vertical line on the plots). The stroboscopic reconstructions exhibit the highest error.

### 4.2 Rotating Chirp

**Fig. 5:**
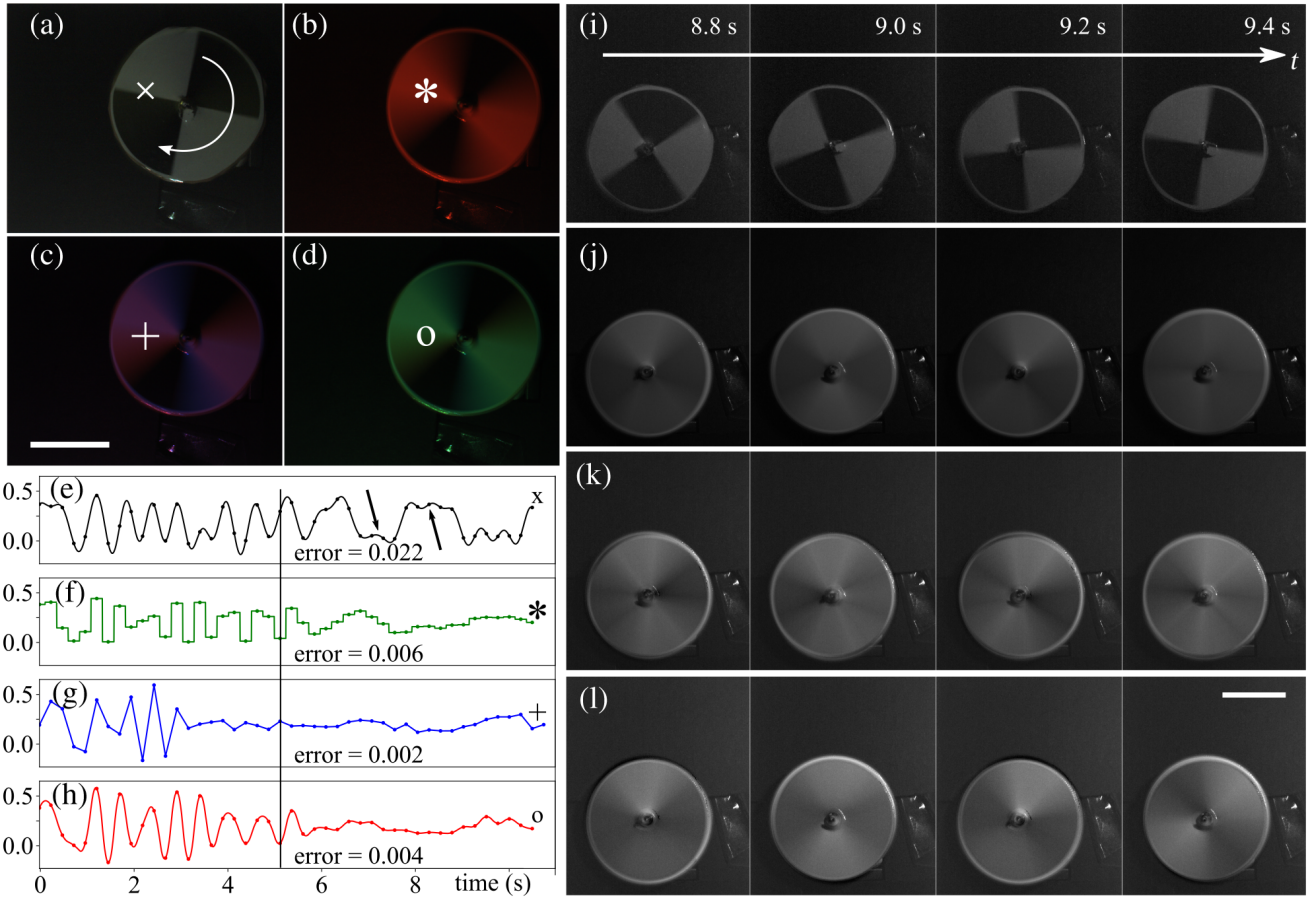
Rotating grid imaged using different sampling methods. (a) Still frame of a rotating grid (arrow indicates direction of rotation, rotation speed was increased over time) under stroboscopic light. (b)–(d) Images of the same grid as in (a), rotating at the same speed, but acquired using B-splines as prefilters, with degrees (b) 0, (c) 1, and (d) 2. (e)–(h) Time profile extracted from the reconstructed image series corresponding to the locations indicated by the symbols x, *, +, and o in (a)–(d), respectively (compare to similar experiment on synthetic data in Fig. 4). Notice that in (e) (stroboscopic case), for t>6, the plot oscillates between the maximal and minimal values (corresponding to white and black in the image) while in (f)–(h) (generalized sampling cases), the values are close to average intensity (gray in the images). (i) Images acquired with the stroboscopic illumination when the grid was rotating fast. Strong aliasing is visible, as between consecutive frames the grid appears to have moved very little, while it has made more than one rotation between each frame. (j)–(l) Reconstructions with B-spline prefilters of degree (j) 0, (k) 1, and (l) 2. Images in (j)–(l) show little to no aliasing: the rotating grid becomes a uniform gray disk, which is consistent with the simulation in Fig. 4. Full movie in Video 1. Scale bars: 2 cm (Video 1 [URL: https://doi.org/10.1117/1.JBO.25.10.106505.1], MP4, 13 MB).

We used a printed black and white grid that we rotated using a stepper motor (Nema 14, Bipolar, Stepper Online, China) controlled by a microcontroller (Arduino Due, Arduino, Italy) through a power driver module (L298N dual H-bridge driver chip, Electronicmodule Store, China). We incrementally increased the grid’s rotation speed by steps, while acquiring images with either stroboscopic illumination or using the illumination functions shown in Fig. 2. The exposure time was set to E=200  ms. We then used the method presented in Sec. 3.1 to reconstruct image series and compared them to those obtained via stroboscopic imaging (Fig. 5). Figures 5(a)–5(d) show acquired images using either stroboscopic imaging (a) or B-spline sampling with degrees 0 to 2 (b)–(d). Figures 5(e)–5(h) show time profiles extracted from the reconstructed image series corresponding to the locations indicated by the symbols x, *, +, and o in (a), (b), (c), and (d), respectively. Figures 5(i)–5(l) show reconstructed images when the grid was rotating at high speed. We can see strong temporal aliasing in (i) as the grid appears to be almost static over consecutive frames, although it has undergone multiple rotations. In Figs. 5(j)–5(l), the rotating grid takes the aspect of a uniform gray disk, with minor intensity variations where aliasing is slightly visible. This experiment shows that the sampling scheme presented in Sec. 3.1 does not allow for perfect sampling, which would be free of aliasing and motion blur. Nevertheless, the prefilter is optimal for the sampling in the bases in which we sample (project) the imaged signal, hence the reconstructions of Figs. 5(j)–5(l) are the optimal representations of the signal in our chosen B-spline bases. In other words, even if our reconstructions are not perfect, they are more reliable than that of Fig. 5(i). Similar to the experiment in Sec. 4.1, we can see that the reconstructions in Figs. 5(f)–5(h) at high speed (right part of the plot, where t>8  s) tend to approximate the average between black and white, which is around 0.2.

Since the ground-truth is not known, we use the energy between the signal and the average gray value between white and black as a proxy for the error at high frequencies, that is, to the right of the vertical line in Figs. 5(e)–5(h), similar to the experiment in Sec. 4.1. As noted by other authors, quantifying aliasing is not a trivial task^22^^,^^23^ and we can only provide a proxy to quantify the error. We see that stroboscopic imaging exhibits the highest error. Also, the two black arrows in Fig. 5(e) show aliasing where the grid appears to be rotating slowly, while it undergoes multiple rotations between two consecutive acquisitions.

### 4.3 Microscopy

We set out to investigate if our method could be implemented in optical microscopy. Specifically, we considered bright field and fluorescence (light-sheet) microscopy. We further investigated the feasibility of using this method for *in vivo* imaging of dynamic processes, specifically, to image the beating heart of zebrafish embryos.

#### 4.3.1 Hardware and parameters setup

For bright-field microscopy (experiments in Sec. 4.3.2), we implemented the illumination scheme with commonly available and cost-effective hardware. We assembled a light source using a 6-LED chip (SLS Lighting RGBWA+UV, Aliexpress, China). We drove the red (λ≈620  nm), green (λ≈525  nm), and blue (λ≈465  nm) LEDs via a microcontroller (Arduino Uno, Arduino, Italy), which we programmed to generate the illumination time-pattern shown in Fig. 2, individually controlling each color. For the LED and camera synchronization, the microcontroller monitored the flash trigger output of the camera. Whenever the trigger signal transitions from low to high state, the microcontroller starts the time sequence of the LEDs for the frame about to be recorded. The LEDs were directly powered by the controller’s outputs without additional power amplification of the signal.

For fluorescence microscopy (experiments in Sec. 4.3.3), we used an implementation of the OpenSPIM light-sheet microscope,^24^ with two lasers (Stradus, Vortran Laser Technology) of wavelengths 488 and 561 nm to generate the excitation illumination light sheet. Again, we used a microcontroller (Arduino Uno) to modulate the laser intensities over time, using pulse-width modulation control on their fast ON/OFF electrical connection to generate the illumination functions in Fig. 2 up to degree 1 (the highest degree achievable with only two lasers).

For both the bright-field and fluorescence experiments, we used a CMOS color camera (Thorlabs DCC3240C, Thorlabs, Germany) with 1280×1024  pixels and a standard RGGB-Bayer filter pattern. We attached the camera to the camera port of our microscope (for both transmission and light-sheet microscopy) consisting of a 20× Olympus water dipping lens (Olympus Plan Fluorite UMPLFLN 20×W) combined with a 180-mm tube lens (Olympus U-TLU-1-2) and terminated by a 0.5× zoom lens (Olympus U-TV0.5XC-3).

For imaging, we embedded 3 dpf zebrafish larvae, anesthetized with 0.1% tricaine (ethyl 3-aminobenzoate methanesulfonate salt, Sigma), in low melting agarose.

#### 4.3.2 Generalized sampling of the beating heart of the zebrafish under transmission microscopy

**Fig. 6:**
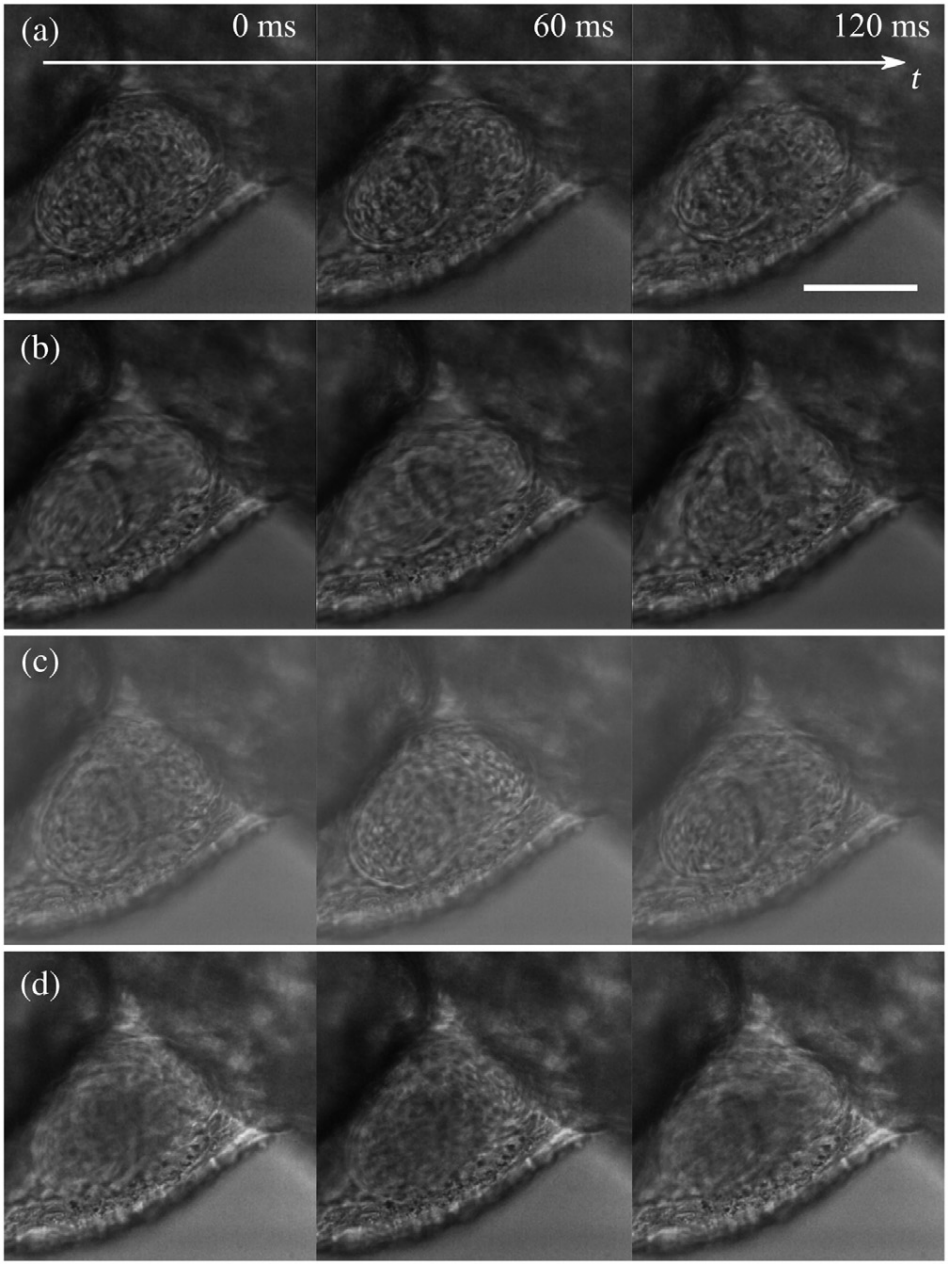
Generalized sampling of a 3-dpf zebrafish beating heart under transmission microscopy. (a) Three consecutive frames of stroboscopic imaging. (b)–(d) Three consecutive reconstructed frames using our method in Sec. 3 for B-spline sampling of degrees (b) 0, (c) 1, and (d) 2. There are no obvious differences between (a) to (d), (a) is slightly sharper than (b) to (d) due to the short light pulse. Full movie in Video 2. Scale bar: 100  μm (Video 2 [URL: https://doi.org/10.1117/1.JBO.25.10.106505.2], MP4, 2 MB).

To illustrate the applicability of our method for biological bright field microscopy, we imaged the beating heart of the zebrafish larva with transmission illumination. We first acquired images of the beating heart using stroboscopic imaging then repeated the acquisition using the generalized sampling method from Sec. 3 with degrees 0, 1, and 2. The exposure time was set to E=60  ms. Figure 6 shows three consecutive images either acquired with a strobed illumination [Fig. 6(a)] or using temporal generalized sampling [Figs. 6(b)–6(d)].

Although the strobed illumination [Fig. 6(a)] produces slightly sharper images, this approach is subject to aliasing at high frequencies. This means that even though the image appears sharper, one cannot trust the observed motions to be representative of the actual motion sequence of the heart. Although the images obtained with our proposed method [Figs. 6(b)–6(d)] exhibit stronger motion blur than those obtained with strobed light, one can be confident that the perceived motion is accurate.

#### 4.3.3 Generalized sampling of the beating heart of the zebrafish on light-sheet fluorescent microscopy

We set out to investigate whether our generalized sampling method is applicable to fluorescence microscopy. To take advantage of the temporally modulated color illumination, we imaged a zebrafish that coexpresses ubiquitous cytoplasmic green fluorescent protein, EGFP, and red fluorescent protein, mCherry.^25^^,^^26^ Taking advantage of the fluorophores being colocalized, we simultaneously computed the inner products in Eq. (8) and used the method of Sec. 3 to perform temporal generalized sampling.

We acquired images of the beating heart of the zebrafish first using stroboscopic imaging then, via the generalized sampling method from Sec. 3, with degrees 0 and 1. The exposure time was set to E=60  ms. Since we had only two lasers and two fluorophores in our system, only generalized sampling up to degree 1 was possible.

**Fig. 7:**
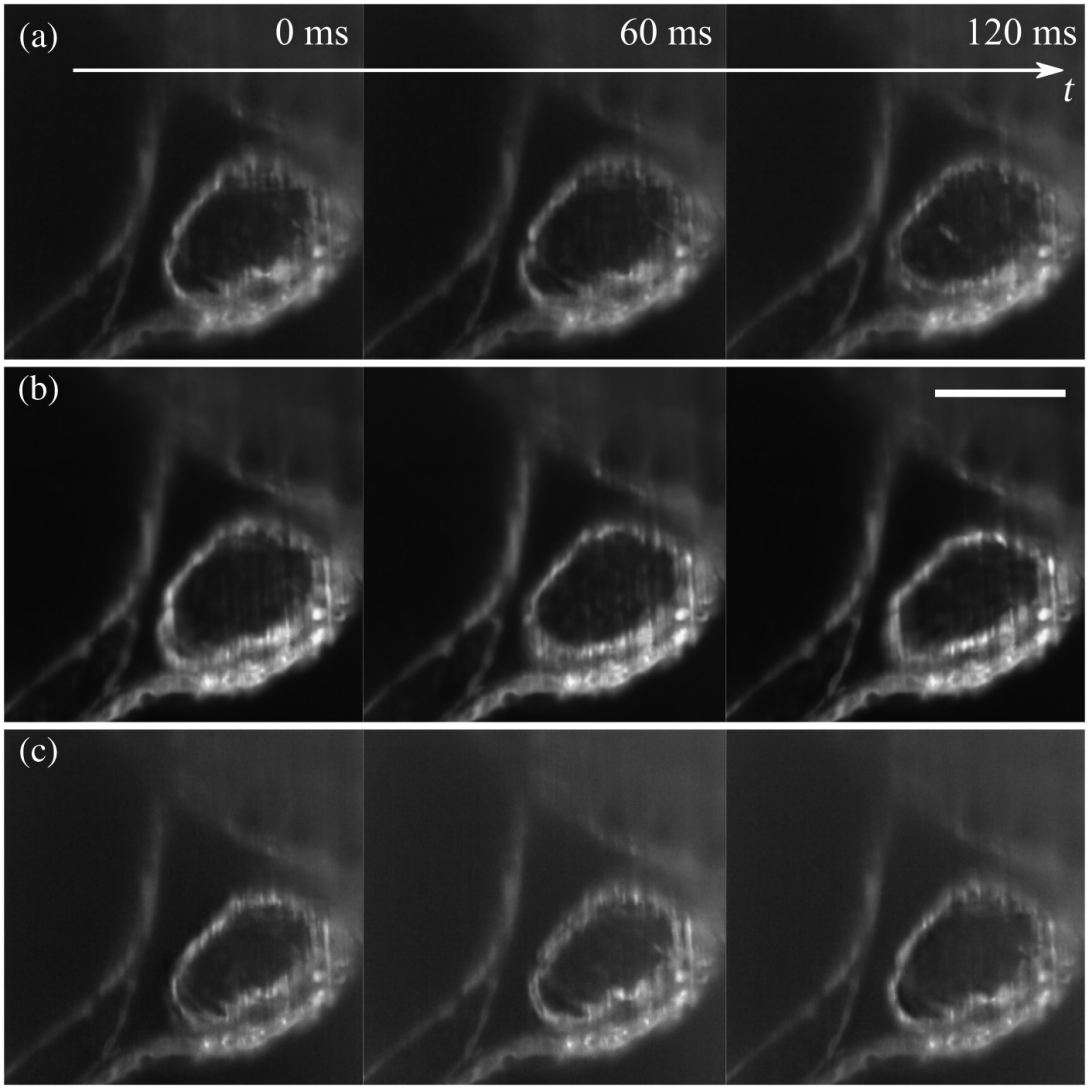
Generalized sampling of a 3-dpf zebrafish beating heart under light-sheet fluorescence microscopy. (a) Three consecutive frames of stroboscopic imaging. (b), (c) Three consecutive reconstructed frames using our method in Sec. 3 for B-spline sampling of degrees (b) 0 and 1 (c). There are no obvious differences between (a) to (c), while the reconstructions in (b) and (c) can be trusted more than (a). Full movie in Video 3. Scale bar: 100  μm (Video 3 [URL: https://doi.org/10.1117/1.JBO.25.10.106505.3], MP4, 1.2 MB).

Figure 7 shows three consecutive images acquired with either the strobed illumination [Fig. 6(a)] or our method [Figs. 6(b) and 6(c)].

Similar to the experiment in Sec. 4.3.2 for transmission microscopy, the strobed illumination produces slightly sharper images. However, the strobed image sequence is subject to stronger aliasing.

## 5 Conclusion

We presented a method to perform temporal generalized sampling in optical microscopy. Our approach allows for the use of implementable prefilters that have finite temporal support and that verify the positivity constraint. Via our proposed spectral multiplexing approach, we could simultaneously compute multiple inner products and, after spectral unmixing, retrieve each individual inner product. Our method leverages the equivalence between basic and dual B-spline representations.^20^ Since our method follows the projection framework of generalized sampling, it offers the prospect of avoiding aliasing despite not using ideal prefilters.

In the experiments of Sec. 4.3, the beating heart of the zebrafish respects the Shannon–Nyquist criterion of our imaging system and lateral motion remains limited, hence our method produces results similar to those obtained via stroboscopic imaging. That is, in this particular case, the imaged sample does not exhibit aliasing when imaged with a stroboscopic imaging system with an image every 60 ms. However, our method increases the trust one can have in the captured videos since an insufficient frame-rate would have resulted in motion blur with our projected method, which would have been visible even in still frames. For biomedical experimentalists, the advantage of using our method is that sharp image sequences are more likely to be devoid of temporal aliasing. Also, while a possible strategy for mitigating the potential risk of aliasing during stroboscopic imaging could be to increase the duration of the pulse width until aliasing disappears (also introducing blurring), this approach would require to actively test for the presence of aliasing, which could be difficult to automatically carry out. Since our method relies on projecting the sampled signal in a predefined basis, the illumination functions are fixed, which may introduce more blurring than necessary in situations where the imaged frequencies are sufficiently low for a strobed approach (Fig. 2).

Higher degree B-splines have a higher approximation power.^27^^,^^28^ Therefore, as high a degree as possible would be preferable. The highest achievable degree, in our experimental setting with an RGB color camera, was 2 since we could compute no more than three simultaneous inner products. While this paper focused on implementing B-spline prefilters, our method of simultaneously computing multiple inner products could be extended to other sensing methods provided the modulation functions are positive and have finite support.

Our experimental implementation relies on a global shutter camera. That is, all pixels share the same integration time and no light is captured by the sensor during the readout (when the image is transferred from the camera to the computer). On the camera we used, this readout time is about 3 ms for images of resolution 500 × 500 pixels. This implies that the partition of unity^16^ condition is not strictly respected. Nevertheless, in practice, this did not visibly affect our method. Some rolling shutter cameras permit continuous exposure of the sensor, where lines are sequentially exposed and transferred to the computer, but they would require additional adaptation to take this exposure sequence into account.
